# Acute symptomatic seizures and hippocampal sclerosis: the major contributor for post-stroke epilepsy?

**DOI:** 10.1007/s00415-022-11254-0

**Published:** 2022-07-07

**Authors:** Patrick Stancu, Pia De Stefano, Maria Vargas, Eric Menetre, Emmanuel Carrera, Andreas Kleinschmidt, Margitta Seeck

**Affiliations:** 1 Neurology Division, University Hospital of Geneva, Geneva, Switzerland; 2 Neuroradiology Division, University Hospital of Geneva, Geneva, Switzerland; 3 EEG & Epilepsy Unit, Neurology Division, Department of Clinical Neurosciences, Faculty of Medicine, University Hospital, University of Geneva, 4, Rue Gabrielle-Perret-Gentil, CH-1211 Geneva, Switzerland

**Keywords:** Hippocampal sclerosis, Stroke, EEG, Acute symptomatic seizures, Provoked seizures, Spikes, Interictal epileptiform discharges, Post-stroke epilepsy, Elderly, Geriatric, Ischemic, Hemorrhagic

## Abstract

**Objective:**

Hippocampal sclerosis (HS) is a prominent biomarker of epilepsy. If acquired later in life, it usually occurs in the context of degenerative or acute inflammatory-infectious disease. Conversely, acute symptomatic seizures (ASS) are considered a risk factor for developing post-stroke epilepsy, but other factors remain unrecognized. Here, we hypothesize that silent hippocampal injury contributes to the development of post-stroke epilepsy.

**Methods:**

We performed a retrospective observational study of patients hospitalized between 1/2007 and 12/2018 with an acute stroke in the Stroke Center of the Geneva University Hospital. Patients were included if they had a documented normal hippocampal complex at onset and a control MRI at ≥ 2 year interval without new lesion in the meantime.

**Results:**

162 patients fulfilled our inclusion criteria. ASS during the first week (*p* < 0.0001) and epileptiform abnormalities in electroencephalography (EEG; *p* = 0.02) were more frequently associated with the development of epilepsy. Hemorrhagic stroke was strongly associated to both ASS and future focal epilepsy (*p* = 0.00097). Three patients (1.8%) developed hippocampal sclerosis ipsilateral to the cerebrovascular event between 2 and 5 years, all with ASS and hemorrhagic stroke.

**Interpretation:**

ASS and epileptiform EEG abnormalities are strong predictors of post-stroke epilepsy. HS develops in a minority of patients after hemorrhagic lesions, leading to focal epilepsy. Prospective studies are required, including follow-up with EEG and if characterized by epileptiform discharges, with MRI, to determine the true frequency of HS and to better understand predictors of post-stroke epilepsy (AAS, stroke type, and HS), and their impact on stroke recovery.

## Introduction

Hippocampal sclerosis (HS) is the most frequent neuropathological lesion underlying drug-resistant temporal lobe epilepsy, usually starting in childhood and found in up to 70% of patients referred for presurgical evaluation for this type of epilepsy [1].

Acquired hippocampal sclerosis in mid-to-late adulthood is rare. It has been mainly described in the context of inflammatory diseases like neurocysticercocis [2], human herpes virus 6 [3], autoimmune limbic encephalitis [4], or aging and neurodegenerative diseases [5, 6], but up to now not as a consequence of acute cerebrovascular disease. Epilepsy is a complication of stroke, however, not as frequent as could be expected. In a prospective study, around 9% of stroke patients suffered from acute symptomatic seizures (ASS), formerly known as provoked seizures, with an almost twofold higher risk in hemorrhagic compared to ischemic stroke [7]. Only 2.5% of stroke patients develop late-onset epilepsy [8]. The term provoked has been replaced by “acute symptomatic”, but both refer to “situation-related” seizures, in contrast to the definition of epilepsy, which requires an enduring predisposition of presenting seizures [9]. Recent evidence suggests that ASS associated with an MRI lesion are not so benign, leading to a relapse risk almost as high as for patients with generalized genetic epilepsy [10].

Acute symptomatic seizures (ASS) have been described repeatedly as risk factor for subsequent seizures [11]. Acute stroke, in particular with bleeding, leads to a strong inflammatory response, which could affect vulnerable structures, like the hippocampus. Secondary HS may occur in this setting and contribute to the development of epilepsy. In the present study, we sought to determine the frequency of post-stroke HS and possible risk factors.

## Methods

We retrospectively identified all patients of interest who were admitted to the Geneva Stroke Center between 1.1.2007 and 31.12.2018. Patients were included if they fulfilled the following inclusion criteria: (1) occurrence of an acute ischemic or hemorrhagic stroke confirmed by cerebral MRI, (2) no previous history of cerebrovascular disease or epileptic seizures, and (3) a second MRI performed at least 2 years after stroke. Reasons to conduct this second MRI were transient neurological deficits or aggravation of existing deficits, to rule out new lesions. Exclusion criteria for all subjects were: (1) patients aged < 18 years; (2) patients with pre-existing atrophy or lesions involving the hippocampus or brain damage; (3) new lesions, e.g., new stroke or tumor, on follow-up MRI. Initial and follow-up MRIs were carried out on a 3-Tesla Philips Ingenia MRI. Clinical data (sex, age, cardiovascular risk factors, and types of stroke) were obtained by review of the medical records.

Acute symptomatic seizures are defined as focal or generalized seizures occurring in the setting of a clearly identifiable, temporally related cause. In the present context, ASS were retained if a seizure occurred within 7 days after the stroke. HS diagnosis was based on established diagnostic MRI criteria, i.e., atrophy and/or signal changes visible in T1- and T2 weighted sequences, with loss of internal architecture and/or hippocampal atrophy (16). Our study was approved by the local ethics committee.

To examine the relation of ASS to EEG findings (spikes vs non-spikes) and the stroke types, we conducted a Chi-square test of independence, including all patients. Regarding comparisons of age and delay of epilepsy between the HS and control group (non-HS in stroke patients with ASS), we used a Mann–Whitney test. A *p* value < 0.05 was considered to indicate statistical significance. Finally, data were expressed as mean ± standard deviation.

## Results

We identified 162 patients (70 female (43%) with a mean age of 78.3 years (± 14.6) who met the inclusion criteria. Based on CT and MRI, 63 (39%) ischemic and 99 (61%) hemorrhagic strokes were diagnosed. Among the hemorrhagic group, 67 (68%) were of primary etiology and 33 (33%) secondary to a vascular malformation, cerebral venous thrombosis, or an ischemic stroke.

Of these patients, 43 presented ASS and 40 of those (93%) were eventually diagnosed with epilepsy after a mean of 3.75 years (± 2.0). Of the 120 patients without ASS only 7 developed epilepsy (*p* < 0.00001). In addition, epilepsy was three times more frequent in the hemorrhagic group (*N* = 37) than in the ischemic group (*N* = 6; *p* = 0.0009). Table 1 shows the clinical data of the patients.

**Table 1:** Demographic and clinical characteristics of the patients included

	Patient 1	Patient 2	Patient 3	Control group (*N* = 159)	*p* value
Sex/age (years)	M/69	M/57	F/63	88 F/ 71 M Mean age: 78.3 ± 14.6	Not significant for both age (*p* = 0.12) and sex (*p* = 1)
Medical history	Cholangio-carcinoma, 50% L carotid stenosis	Basilar migraine	Unrevealing	150 with relevant medical history	*p* = 0.22
Acute symptomatic seizures (ASS)	Yes	Yes	Yes	43 (27%)	*p* = 0.068
Presence of epilepsy	yes	Yes	yes	40 (25%)	*p* = 0.0005*
Delay of epilepsy onset after stroke	5 years	2 years	3 years	Mean: 3.17 ± 1171.2	*p* = 0.2937
MRI	L fronto-parietal stroke	L parieto-temporal stroke	L fronto-parietal stroke	56 strokes in the MCA, 6 cerebral venous sinus thrombosis, 28 PCA, 28 ACA, 25 basal ganglia hemorrhage, 7 watershed, 9 cerebellar	Stroke localization: *p* = 0.13 Lateralization of stroke (L VS R): *p* = 0.62
EEG	L parieto-temporal spikes	L fronto-temporal slowing	L parietal slowing	72 with routine EEG; 54 presented IEDs	*p* = 0.06
Type of stroke	hemorrhagic	hemorrhagic	hemorrhagic	63 ischemic/96 hemorrhagic	*p* = 1
Follow-up MRI	L HS	L HS	L HS	No HS	NA

*Mann–Whitney test, HS vs control group

Only 72 (45%) of all patients underwent routine EEG in the acute phase after stroke. In 54 (75%) patients, epileptogenic discharges were noted, and of those, 40 (75%) developed epilepsy. Of the remaining 18 patients without interictal epileptiform discharges, 8 developed epilepsy (44%; *p* = 0.021). Hemorrhagic stroke was strongly associated with ASS and a future diagnosis of focal epilepsy (*p* = 0.00097).

**Fig. 1:**
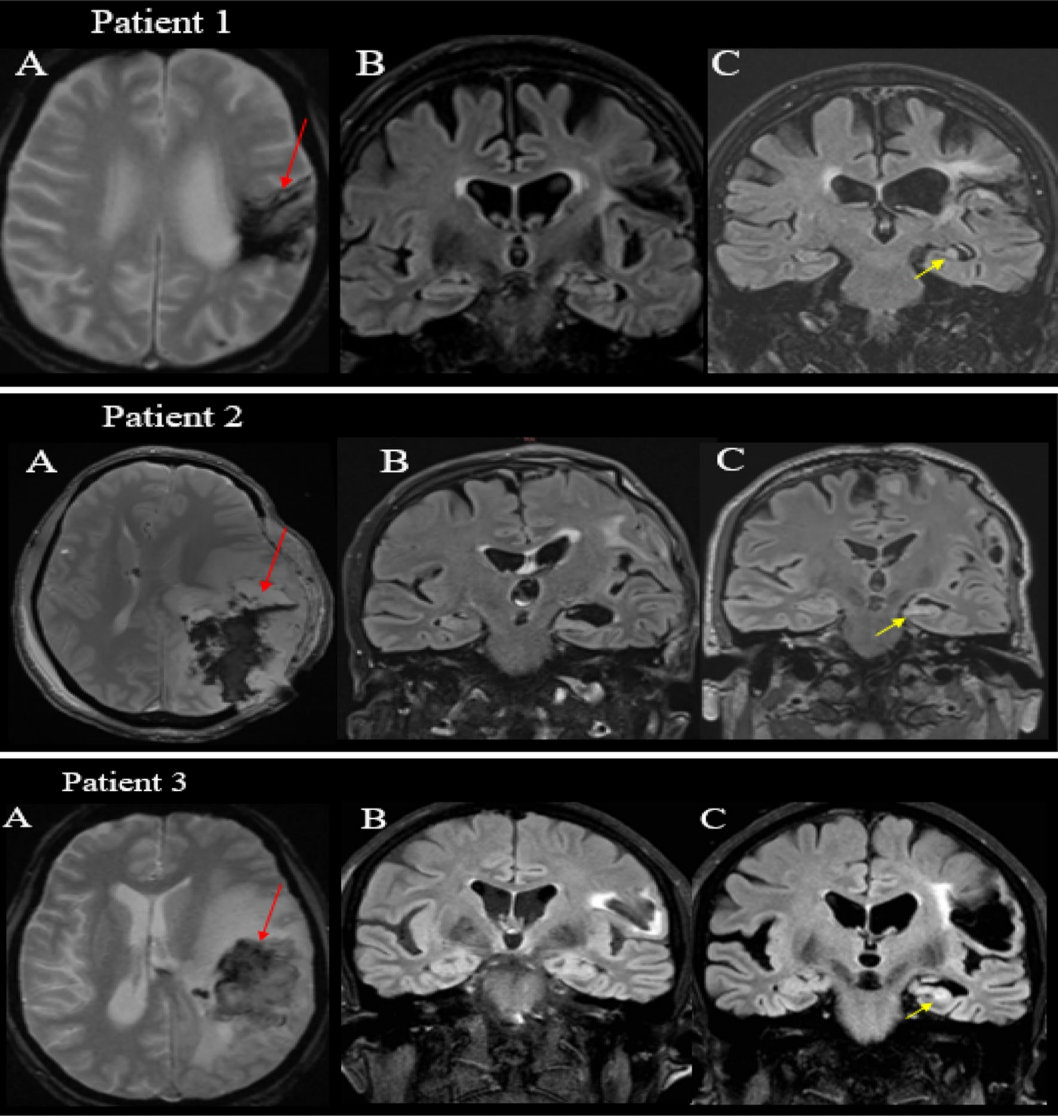
Cerebral MRI from the initial insult to the hippocampal sclerosis in patients 1, 2, and 3. **A** Axial SWI-MRI showing the acute hemorrhagic lesion (red arrow). **B** Coronal MRI FLAIR showing symmetric hippocampi at stroke onset. **C** Coronal MRI FLAIR showing hippocampal sclerosis after a minimum of 2 years (yellow arrow). *FLAIR* Fluid-attenuated inversion recovery, *SWI* Susceptibility weighted imaging

Three patients (1.8%; 2 female) developed hippocampal sclerosis, visible in the follow-up MRI but not in the initial one at admission for stroke (Table 1, Fig. 1). Follow-up (F/U) MRI was performed at a mean of 38 (± 31) months after onset. All three patients showed increased signal in FLAIR and T2 sequences of the left hippocampus and all suffered from epilepsy compared to 25% in the control group (*p* < 0.00086). Type of stroke, i.e., ischemic vs hemorrhagic, sex ratio, and mean age did not differ between the 3 and 159 patients without HS. All three cases had HS ipsilateral to the cerebrovascular injury, and all three showed increased signal in FLAIR and T2 sequences in the left hippocampus.

## Discussion

Our retrospective study suggests that acute hemorrhagic stroke combined with acute symptomatic seizures leads to epilepsy and in some cases to secondary hippocampal sclerosis, as determined in follow-up MRIs.

Primary or secondary hemorrhagic stroke is more frequently associated with epilepsy as shown by the present results and observations from other centers. A 2.5 × higher risk is reported [12], possibly due to the presence of ASS. Previous studies reported ASS in 10–16% of patients with intracranial hemorrhages, including hemorrhagic transformation of an ischemic stroke vs. 2% and 4% of patients with ischemic strokes [13, 14]. Our results confirm the high risk to develop epilepsy when ASS occur after stroke [12, 15]. Moreover, results from our lab and of other groups indicate that the presence of epileptogenic discharges in the EEG strongly suggests the presence of focal epilepsy in ASS-patients [16, 17].

A small subset of patients, all with hemorrhagic stroke, developed hippocampal sclerosis ipsilateral to the insult. To the best of our knowledge, this is the first study on acquired HS in stroke. The exact mechanism of HS are still unknown and histopathological studies of HS have shown that neuronal loss is mainly seen in the CA1 subregion and subiculum, regardless of etiology [18, 19]. In stroke patients, focal hypoxia or failure of cerebral autoregulation could be incriminated. Secondary vasogenic edema may result in HS as noted in patients with renal insufficiency and hypertension [20, 21]; however, we did not find evidence for these complications in our patients. The proximity of the hemorrhagic perisylvian lesion and the localization of the ipsilateral HS are highly suggestive of local inflammatory process affecting the hippocampus nearby [22, 23].

All hippocampal changes occurred on the left side. While this could be a chance finding, given the low number of cases, there is evidence that the left hippocampus is more vulnerable than the right. Overall, left HS is found more frequently reported than right HS in childhood-onset temporal lobe epilepsy [24]. In Alzheimer’s disease, the left mesial temporal structures are often affected first [25]. The left hemisphere develops later than the right [26], correlating with the earlier acquisition and bilateral representation of visuospatial functions vs language functions, which are more likely to be impaired if a lateralized insult occurs. We are not aware of a systematic analysis of the side of epileptiform discharges in stroke patients, to confirm or refute the hypothesis of a particular vulnerability of the left hemisphere or, more specifically, left temporal structures.

Our study has several limitations. Hemorrhagic strokes make up about 10–20% of strokes, but in our sample, 61% were of hemorrhagic nature, which indicates a strong sampling bias. However, we were interested in the development of post-stroke HS, requiring an observation period of at least 1–2 years and a control MRI. Our inclusion criteria may have led to an overrepresentation of patients with possible or manifest epilepsy. We hypothesize that new symptoms motivating the control MRI and routine EEG were often short seizures in a large number of patients, which would also explain the high amount of epileptiform discharges (73%) in the routine EEG of our cohort.

Large prospective studies are mandatory to determine the true frequency of epilepsy and HS, the predictive value of early EEG findings, and the role of ASS for developing post-stroke epilepsy. Such studies could help to better understand the relationship between ASS, post-stroke epilepsy, HS, and finally also the cognitive and neurological consequences of epilepsy and/or HS for stroke patients. In a recent prospective study with close follow-up, 25% of the 151 patients developed epilepsy within 2 years [27], which suggests a much higher incidence (and underdiagnosing) of epilepsy in stroke patients.

An acute symptomatic seizure is defined as a clinical seizure occurring at the time of a systemic insult or in close temporal association with a documented brain insult, during the first 7 days [9]. The term ASS signifies a transient disturbance with low risk of recurrence if the underlying condition is successfully treated. This definition may not be adequate in stroke where ASS appears to indicate onset of epilepsy in the majority of patients, symptomatic to a lasting lesion. The prognosis is different, if seizures are related to focal epileptiform discharges vs unspecific changes in the EEG, or if they occur during the 1st day with severe hyponatremia versus the 7th day post-stroke with normal blood test results. Worsening of stroke deficits due to focal, recurrent seizures has been described, and in some patients, they were irreversible [28, 29]. A more aggressive approach regarding work-up of “ASS”, i.e., with routine EEG, laboratory exams, and sleep EEG (if routine EEG is unrevealing), is highly desirable for modern stroke care in the twenty-first century.
